# PROTOCOL: Body‐worn cameras’ effects on police officers and citizen behavior: A systematic review

**DOI:** 10.1002/cl2.1043

**Published:** 2019-09-19

**Authors:** Cynthia Lum, Christopher S. Koper, David B. Wilson, Megan Stoltz, Michael Goodier, Elizabeth Eggins, Angela Higginson, Lorraine Mazerolle

**Affiliations:** ^1^ Department of Criminology, Law and Society George Mason University Fairfax Virginia; ^2^ School of Social Science University of Queensland Brisbane Queensland Australia; ^3^ School of Justice Queensland University of Technology Brisbane Queensland Australia

## 1 BACKGROUND

### 1.1 The problem, condition or issue

Body‐worn cameras (BWCs) are one of the most rapidly diffusing technologies in policing today, costing agencies and their municipalities millions of dollars. This adoption has been propelled by highly publicized events involving police use of force or misconduct, often linked to concerns of racial and ethnic discrimination (see general discussions by Braga, Sousa, Coldren, & Rodriguez, 2018; Lum, Stoltz, Koper, & Scherer, 2019; Maskaly, Donner, Jennings, Ariel, & Sutherland, 2017; Nowacki & Willits, 2018; White, 2014). In culmination, these contexts fostered enough public and political will to generate an urgent call for BWCs. This demand was matched with a prepared supplier; technology companies had already been developing both BWCs and other similar surveillance devices (e.g., in‐car cameras, license plate readers, and closed‐circuit televisions). In the United States, an estimated 60% of local police departments have fully deployed BWCs (Hyland, 2018). Similar widespread testing, piloting, and adoption of BWCs have also occurred in the United Kingdom, Australia, and Europe.

Given the rapid and widespread adoption of BWCs, their significant costs, and their potential impacts on law enforcement agencies and the communities they serve, an important question for practitioners, government officials, and researchers is whether the cameras effectively achieve the expectations of them. In their narrative review of empirical BWC research, Lum et al. (2019) suggest there may be equivocal answers to the question of BWC effects. This systematic review of BWCs reviews and synthesizes existing research to examine these concerns.

### 1.2 The intervention and how it might work

BWCs are small surveillance and information technologies that law enforcement officers wear on their clothing or glasses. These cameras can be turned on manually or automatically based on a variety of procedures, policies, rules, or prompts that are usually determined by an agency, government, or other municipal oversight groups. When operating, BWCs record interactions, activities, and events from an officer's vantage point. Some cameras can also record a small time period before and after the cameras are activated to capture a wider time frame around events that officers choose to record.

Given their recording capabilities, BWCs are believed to serve a number of functions. Because of the context in which they were adopted, BWCs are intended to document interactions between police and citizens to increase transparency and accountability in the investigation of police misconduct. It is also believed that BWCs can reduce the use of force by officers, deter civilian assaults on officers, and have a mitigating effect on complaints against officers. The primary mechanisms behind how BWCs might work to achieve some of these outcomes is deterrence and self‐awareness. BWCs are theorized to have a deterrent effect on excessive use of force or unlawful actions by officers because officers become self‐aware that they are being recorded by their camera or other cameras worn by fellow officers. As noted by Ariel et al. (2017) in their discussion of the application of deterrence and self‐awareness theories to BWCs: “It is hypothesized that the self‐awareness that arises when we are aware of being watched/filmed drives us to comply with rules/norms, primarily because of the perceived certainty of punishment.” (p. 297) BWCs may also similarly serve to deter individuals that officers encounter. For example, civilians may see the BWCs (or be alerted to them verbally by officers) and then moderate their behavior accordingly.

Existing research both supports and challenges the deterrence and self‐awareness hypothesis of BWCs. As Lum et al.’s (2019) narrative review has found, early research seemed to show that BWCs reduced the use of force by officers. More recent findings, however, have been mixed. The hypothesized self‐awareness imposed on officers by the cameras may also affect their use of arrests, citations, and proactive activities, which may have further implications (both positive and negative) for crime prevention and police‐citizen relations. However, research in this area is also ambiguous. Some studies indicate arrests or proactive activities increase, while other studies indicate these activities do not change or even decrease. Further, Lum et al. (2019) also point out that while BWCs seem to reduce complaints against officers, it is unclear whether this reduction is due to the changes in officer behavior (which would indicate a deterrent effect on officers); changes in citizen behavior (which may also indicate a deterrent effect on civilians); or changes in reporting practices by officers and citizens (which does not necessarily point to a deterrent effect). A further complication: McClure et al. (2017) found that many citizens who interact with police cannot even remember whether officers were wearing BWCs (see also White, Todak, & Gaub, 2017).

An additional challenge to understanding BWC effectiveness is that survey research has shown there is an incongruence in the expectations that police and community members have for BWCs (Lum et al., 2019). The meaning of “effectiveness” for the police may not be the same as what “effectiveness” means for civilians. The police may view cameras as effective when they protect officers from frivolous complaints and assaults and when they strengthen officers’ ability to arrest and prosecute offenders. Civilians, in contrast, may judge BWC effectiveness by whether cameras provide greater accountability and transparency for officer actions and protect the public against excessive use of force and officer misconduct. And, while some of these effects can be explained from a deterrence perspective, other effects are purely technical or organizational. For example, BWCs are also believed to improve investigations and case clearances. Here, the theoretical mechanism is straightforward: if a crime or an important piece of evidence is captured on an officer's BWC, it can be used to more effectively prosecute offenders. Organizational effects may include the effects of BWCs on training, which delves into the realms of educational theory (i.e., visual aids for learning may create better retention of experiential knowledge for application in the future).

### 1.3 Why it is important to do this review

Because the rapid adoption of BWCs was driven by public protest, law enforcement concerns, government funding, and the development and marketing of portable video technology, it should not be any surprise that BWCs were quickly adopted in a low‐research environment (Lum, Koper, Merola, Scherer, & Reioux, 2015). The importance of scientific inquiry about police technologies like BWCs, however, cannot be overstated. If law enforcement—and ultimately, citizens—intend to invest heavily in BWCs, then BWCs should produce the outcomes we expect of them. Unfortunately, however, researchers have consistently found that police technologies may not lead to the outcomes sought and often have unintended consequences for police officers, their organizations, and citizens (Chan, Brereton, Legosz, & Doran, 2001; Colton, 1980; Koper, Lum, Willis, Woods, & Hibdon, 2015; Lum, Hibdon, Cave, Koper, & Merola, 2011; Lum, Koper, & Willis, 2017; Manning, 2008; Orlikowski & Gash, 1994). Without the results of rigorous research and evaluation, law enforcement leaders are left to rely on best guesses, hunches, notions about “craft,” and “group think” about the impact of technologies like BWCs (see discussion by Lum & Koper, 2017). Research knowledge about technologies, if mind, can help law enforcement agencies better anticipate unintended consequences from technologies, optimize their use of already acquired technologies, or decide whether to invest in specific technologies.

The first review of BWCs was conducted by White (2014), who discovered that only five evaluation studies had been completed as of September 2013, even though almost a third of U.S. agencies had already adopted BWCs and widespread adoption was being planned in the UK and Australia. In other words, agencies had already begun rapidly adopting BWCs without clear knowledge about whether the technology could deliver on the high expectations that many had for it (i.e., to increase police accountability, reduce the use of force, reduce disparity, and improve community relationships). Fortunately, researchers have taken a major interest in studying BWCs in the last 5 years and have tried to keep up with its rapid adoption. For example, by November 2015, Lum et al. (2015) found that completed studies about BWCs had grown to more than a dozen, with 30+ additional studies underway. Most of the studies included in both White's and Lum et al.’s reviews were focused on BWCs’ impacts on officer behavior, as measured by complaints and use of force, and on officer perceptions about BWCs. Maskaly et al. (2017), in a review of police and citizen outcomes more specifically, found 21 empirical studies as of January 2017, which led them to conclude that police are generally receptive to BWCs, and that the cameras can exert positive effects on police behavior.

In their most current and comprehensive narrative review of BWCs that included all empirical studies found or accepted for publication through June 2018, Lum et al. (2019) discovered approximately 70 published or publicly available studies of BWCs that contained over 110 sub‐studies examining various outcomes and aspects of BWCs. They grouped these studies into six topical categories: (a) the impact of BWCs on officer behavior; (b) officer attitudes about BWCs; (c) the impact of BWCs on citizen behavior; (d) citizen and community attitudes about BWCs; (e) the impact of BWCs on criminal investigations; and (f) the impact of BWCs on law enforcement organizations. Many of these studies were outcome evaluations, an unusual development in technology research where outcome evaluations are often lacking.

In their narrative review, Lum et al. (2019) concluded that although it appears that many agencies and officers support BWCs, BWCs have not consistently had the effects intended by either police officers or community members. They argue that anticipated effects may have been “overestimated” and that behavioral changes in the field may be “modest and mixed.” They also discuss that while study findings have indicated that complaints have declined in many evaluations of BWCs, it is unclear *why* the decline occurs and whether the actual interactions or relationships between the police and the public have improved. There are some outcomes that have not been investigated—in particular, the impact of BWCs on racial and ethnic disparities in policing outcomes, the alleviation of which was a major reason for some communities to push for BWC adoption. At the same time, Lum et al. state that BWCs will continue to be adopted by police agencies, which makes the production and synthesis of rigorous research even more essential to this policy area.

Lum et al. (2019) did not conduct a systematic review and meta‐analysis of BWC studies, and therefore many questions remain about what we can conclude about the impacts of BWCs from the existing research. Perhaps findings might be conditioned by the quality of research studies and designs, the location and timing of evaluations conducted, or even by the groups involved in the research (much of the BWC research has been clustered amongst groups of researchers at specific universities). A major concern with BWC outcome evaluations has been the extent of contamination between the treatment and control groups, as well as how outcomes are measured. Lum et al. (2019) acknowledge the importance of a systematic review to parse out these important aspects of studies.

## 2 OBJECTIVES

The primary objective of this review is to synthesize and explore the evidence on the impacts of BWCs on several outcomes of interest to police, policymakers, and the wider community. Specifically, given the existing research found by Lum et al. (2019), this review will focus on examining two categories of effects of BWCs:

The impact of BWCs on officer behaviors, as measured by officer use of force, complaints, arrest and citation behavior, and proactive activities. We note that changes in citizen complaints might also be a measure of civilian behavior, as discussed above. However, for this review, it will be used as a measure of officer behavior.
The impact of BWCs on civilian behaviors, as measured by community members’ compliance with police commands (to include resisting arrest or assaults against officers). Studies examining this type of impact may also include evaluations of whether BWCs deter criminal or disorderly conduct of community members. Additionally, some studies have examined citizen willingness to call the police (either as a victim or witness) or cooperate in criminal investigations.

The second objective of this review is to explore explanations for variations in effect sizes and directions of effects that are likely to be found across studies. Explanations could be due to variations in the location, context, quality, or characteristics of research studies. Toward this end, a number of post‐hoc moderator analyses will be run. For example, there may be differences in findings between experimental and quasi‐experimental studies, although what the difference might be is unclear. While evaluations in criminal justice have indicated that experimental studies may find smaller effects, this is not the case in other related areas. Another possible moderator might be the study's country. Findings may differ, for example, between BWCs studies conducted on U.S. jurisdictions compared to other jurisdictions because of the context of BWC adoption in the U.S. Another possible post‐hoc moderator analysis could be done on the author group involved in the study (see example in Petrosino et al., 2014). A sizeable portion of BWC research has been conducted by research groups at Arizona State University and also Cambridge University. Yet another possible post‐hoc moderator analysis that might be examined is the year of BWC adoption by agencies. Although BWCs impact the specific agency examined, the use and public discourse around BWCs have been widespread. Evaluations of early adopters may yield different results than evaluations of later adopters. Again, these moderator analyses and others will be post hoc, given that there is little theory or empirical research to predict outcomes.

Overall, the goal of the review will be to provide practical information to police agencies, municipalities, governments, and citizens as to whether to adopt BWCs or to more carefully consider what BWCs might do for them. As with Lum et al.’s narrative review, this review should also serve as a starting point for debate and conversation about incongruent expectations of BWCs among officers and civilians. Because technologies often lead to unintended consequences for both agencies and the communities they serve, research syntheses can also help to highlight the possibility of such consequences and help agencies and communities plan for potential future impacts of BWCs.

## 3 METHODOLOGY

Given the two objectives discussed above as well as variations in outcomes measured, this systematic review will be organized into two sections (impacts on officer behavior and impacts on civilian behavior). The review may be further broken down into different subareas depending on the outcomes measured (see Gill, Weisburd, Telep, Vitter, & Bennett, 2014, who took a similar approach in their Campbell systematic review of community‐oriented policing).

### 3.1 Criteria for including and excluding studies

#### 3.1.1 Types of study designs

Both experimental and quasi‐experimental designs will be included in this review. Experimental designs will be eligible if treatment is randomly assigned to the units of analysis. BWC studies have used various units of analysis, from individual officers to officer‐shift combinations, to investigative cases. Thus, eligible experimental designs will include those in which BWCs are randomly assigned to officers, officer‐shifts, or other units.

Quasi‐experimental studies will also be eligible for this review if a similar comparison group is evident in the study. Study authors may develop a comparable comparison group using propensity score or other matching techniques achieved through the use of statistical controls. Matching may be at the individual level of at the group level. Statistical control methods can include regression, analysis‐of‐covariance, and propensity score matching, among others. Use of a statistical control method is sufficient for inclusion; we will not exclude studies based on a subjective assessment of the quality of the statistical controls. Rather, any quasi‐experimental design that controls for possible explanations for BWC outcomes, such as officer characteristics (race, gender, age, time in service, rank, etc.) or civilian or event characteristics (race, gender, age, situation, the reason for the stop, etc.) will be eligible. Quasi‐experimental designs that do not have a comparison group or do not use the above methods to achieve comparability are not eligible for inclusion in this review.

#### 3.1.2 Types of participants

Given the objectives outlined above, the population of interest is law enforcement officers and civilians. However, it should be noted that the units of analysis could vary, to include officers, groups of officers, officer‐shift combinations, and non‐law enforcement personnel (community members, citizens, etc.).

In the original title for this protocol, we also listed the “police organization” as being impacted by BWCs. However, given that scarcity of experimental or quasi‐experimental research on BWC impacts on organizations, we will not be pursuing this area of BWC research. Discussions of the scarcity of research in this area have been explored by Lum et al. (2019).

#### 3.1.3 Types of interventions

Studies that examine the use of BWCs by law enforcement officers will be eligible for this review. Excluded are studies that focus solely on the use of BWCs for interrogations in an interrogation room within the police agency.

#### 3.1.4 Types of outcome measures

Given the possible impacts of BWCs on a wide variety of outcomes as discussed by Lum et al. (2019), multiple outcomes measures are considered in this review. Thus, subanalyses will be conducted and organized similarly to Gill et al.’s (2014) systematic review of community‐oriented policing. Specifically, the following outcomes will be examined within each of the three general categories of studies outlined above:

1. Officer behavior
  - officer use of force
  - complaints against officers
  - arrest and citation behavior
  - proactive activities
2. Civilian behavior
  - compliance with police commands as measured by resisting arrest
  - assaults against officers (which may overlap with 2a, above)
  - criminal or disorderly conduct
  - willingness to call the police or cooperate in an investigation

We note that in the Title submitted for this review, we suggested the possibility of examining officer and citizen attitudes towards BWCs for this review. As Lum et al. (2019) discovered, survey research reflects a significant portion of the empirical research on BWCs. However, we do not include officer or citizen attitudes toward BWCs in this systematic review for the following reasons: First, most of this research is neither experimental nor quasi‐experimental, but descriptive. A systematic review of such research would require a separate protocol and review. Second, that literature is not focused on the impact of BWCs on behavior, but rather perceptions of BWCs generally, or perceived beliefs about behaviors. The focus of this systematic review is on measured outcomes, rather than perceived ones. Finally, Lum et al. (2019) have already conducted a preliminary review of this survey research and are currently undertaking a more in‐depth exploratory review of this research outside of the Campbell framework.

We also note that in the Title submitted for this review, we suggested the possibility of examining case or investigative outcomes. For example, Lum et al. (2019) found two experimental studies that examined the impact of BWCs for domestic violence case outcomes (Morrow, Katz, & Choate, 2016; Owens, Mann, and Mckenna, 2014). However, we decided to exclude this category of studies from this review. There is a large body of research that examines the impact of videotaping more generally on interrogations and interviewing of suspects, witnesses, and victims, and the use of videos and court outcomes. Given that these outcomes do not specifically focus on the impact of cameras on officer and citizen behavior and given the overlap of this area with other unrelated areas (investigatory effectiveness using video technologies), we excluded this area of research from this review.

#### 3.1.5 Duration of follow‐up

The expected effects of BWCs are immediate. That is, they are presumed to have an effect while they are being used. As such, the outcomes are measured concurrently with the intervention and no follow‐up period is needed in assessing their effects. Some studies do measure the longer‐term effects of BWCs, but in these studies, the BWCs are still in use. While we do not expect to find studies that measure effects at a follow‐up period after the BWCs are no longer in use, if such a study is located during the search and screening process, it will be included in the review.

### 3.2 Search strategy and screening process

The search for BWC research will be led by the Global Policing Database research team at the University of Queensland (Elizabeth Eggins and Lorraine Mazerolle) and Queensland University of Technology (Angela Higginson). The University of Queensland is home to the Global Police Database (see http://www.gpd.uq.edu.au), which will serve as the main search location for this review. As detailed by Higginson, Eggins, Mazerolle, and Stanko (2015), the GPD “is a web‐based and searchable database designed to capture all published and unpublished experimental and quasi‐experimental evaluations of policing interventions conducted since 1950. There are no restrictions on the type of policing technique, type of outcome measure or the language of the research” (p. 1). The GPD is compiled using systematic search and screening techniques, which are reported in the Higginson et al. (2015) and summarised in Appendices A and B. Broadly, the GPD search protocol includes an extensive range of search locations to ensure that both published and unpublished research is captured across criminology and allied disciplines.

To capture studies for this review, we will use BWC specific terms to search the GPD corpus of full‐text documents that have been screened as reporting a quantitative impact evaluation of a policing intervention. Specifically, we use the following terms to search the title and abstract fields of the corpus of documents published between January 2004^1^ and December 2018:

camera⁎video⁎ORBWC⁎ORBWV⁎

The results of this search will then be processed using a two‐coder system. The abstract for each study found from the search above will be examined by two coders, who will separately determine whether a study is “potentially eligible” for further full‐text review given this protocol's criteria; “not eligible” for further full‐text review; “unclear” (the coder could not make a determination given the information given); or a “relevant review” (the article is not a study, but should be flagged as a relevant review of studies. The codes from both coders will be reviewed for differences by a principal investigator (Lum or Koper). Each difference will then be discussed and if needed, mitigated by a third coder. Studies with differences that persist and cannot be mitigated (specifically if one coder continues to believe a study is “potentially eligible”) will be retained and the full text of the study will be examined in the next screening process.

After reviewing the initial abstracts from the GPD, the research team will also examine whether any relevant studies from Lum et al. (2019) and from the Bureau of Justice Assistance's BWC Toolkit Resources^2^ were missed in the GPD search. Both sources of information contain comprehensive research collections on the impacts of BWCs on officer and citizen behavior and may contain studies relevant for this review.

Once studies are determined by at least one coder to be “potentially eligible”, the full‐text document of each study will be obtained and examined separately by two coders for eligibility according to the “Criteria for Including and Excluding Studies” as described above. Studies must satisfy these criteria in order to be included in the systematic review. If the coders differ in their assessment, a third coder will be used to examine the study for eligibility. If a study continues to draw debate, other coders and expert may be consulted to determine its eligibility for the systematic review.

### 3.3 Criteria for determination of independent findings

The primary unit‐of‐analysis for this review will be a research study defined as a distinct sample of study participants involved in a common research project. Multiple reports (e.g., publications, technical reports, etc) from a common research study will be coded as a single study. Stated differently, a research study will only be treated as unique if the study sample does not include study participants included in any other coded study. Multiple effect sizes will be coded, if possible, from studies when multiple outcomes are analyzed. Statistical independence will be maintained or modeled in all statistical analyses.

The choice of outcomes in this review will be prioritized. For example, while there are many different types of use of force (e.g., hands only, nonlethal instruments, firearm use) and complaints (i.e., complaints of rudeness, service delivery), we will select the most general measure of use of force or complaints measured (i.e., counts of reports of use of force or complaints generated). Additionally, there are many different types of crimes and infractions that may receive arrest and citations, but only the most general measure of arrest and citation will be measured (i.e., “all arrests” or “all citations”). Similarly, for non‐police civilian behaviors, the more general behavioral categories will be measured (i.e., “resisting arrest,” “assault on officers,” etc). With regard to officer proactivity, a decision may need to be made as to whether to examine the overall levels of proactivity or specific types of proactivity (i.e., stop‐question‐and‐frisks, traffic stops, pedestrian stops, problem‐solving, community policing, etc). As Lum et al. (2019) discuss, not all proactive activities are viewed similarly by either the police or community members, and may need to be parsed out during the analysis to examine BWCs impacts on different types of proactive police behaviors. Finally, for the impacts of BWCs on investigative case files, general categories will be used, including “arrest” or “conviction.”

### 3.4 Details of study coding categories

Per Campbell policy, all studies will be double‐coded. Detailed coding categories and instructions are presented in the Appendix C. Coding will include information on the nature of the BWC use, comparator condition, contextual features of the agency, method and design features, dependent measures and effect sizes for the above outcomes, and risk‐of‐bias indicators. Data will be maintained in a relational database (MySQL) with coding forms developed in LibreOffice Base (similar to MS Access).

### 3.5 Statistical procedures and conventions

Based on prior work by Lum et al. (2019), we expect to find a sufficient number of studies to conduct a meta‐analysis for the three broad outcomes described above. However, given the various outcomes and study designs that are likely to be found, a variety of approaches to calculating effect sizes will have to be used, as described by Lipsey and Wilson (2001). Various effect sizes will be then converted to Cohen's d except for outcomes that are more naturally measured dichotomously, in which case the odds ratio will be used. Calculation techniques as described by Lipsey and Wilson and the online effect size calculator developed by David Wilson will be employed.

A meta‐analysis will be conducted using random‐effects models estimated via full‐information maximum likelihood. Primary analyses will be performed using Stata packages developed by David B. Wilson and available at http://mason.gmu.edu/~dwilsonb/ma.html. The robust standard error method of modeling statistical dependences will be implemented with the Stata package robumeta (see http://www.northwestern.edu/ipr/qcenter/RVE‐meta‐analysis.html for details). Moderator analyses of a single categorical variable will be fit using the analog‐to‐the‐ANOVA method, also under a random‐effects model. Moderator analyses of continuous moderators or multiple moderators will be conducted with meta‐analytic regression methods, also under a random‐effects model. Results will be presented separately for experimental (randomized) and quasi‐experimental designs, although these may be combined in moderator analyses.

Publication‐selection bias will be assessed in three ways. First, analyses will compare the results from published and unpublished reports. Published documents will include peer‐reviewed journal articles, books, and book chapters. All other report forms, such as theses, technical reports, government and agency reports, will be considered unpublished. Second, we will perform a trim‐and‐fill analysis on the major outcome categories. Third, we will visually inspect a funnel plot on the major outcome categories.

Sensitivity analysis will be conducted if needed, based on initial findings. As already discussed, moderator analysis will be conducted for this review, and can include (but is not limited to) the following:

- Whether the study was done inside or outside of the United States (where most BWC research has been conducted)
- Whether research was conducted by specific dominant research teams that conduct BWC research
- Type of research design (i.e., experimental versus quasi‐experimental)
- Publication type (published versus unpublished)
- Evaluation on earlier or later adopters

We do not plan to include qualitative research in this systematic review, except as to provide context for interpreting results.

## ROLES AND RESPONSIBILITIES

- Content: Lum, Koper, Wilson, Stoltz, and Goodier
- Systematic review methods: Wilson, Lum, and Koper
- Statistical analysis: Wilson, Lum, and Koper
- Information retrieval: Eggins, Higginson, Mazerolle, Stoltz, and Goodier

## SOURCES OF SUPPORT

This systematic review is supported by funding from the Laura and John Arnold Foundation

## DECLARATION OF INTERESTS

None of the authors have been involved in an evaluation of BWCs in a police agency. We also have not received any payment or have had any contractual agreements with any technology company who manufactures or sells BWCs, or who profits from sales of BWCs to law enforcement agencies.

## PRELIMINARY TIMEFRAME

**Table:** 

1/31/2019	Submission of Protocol
3/31/2019	Completion of the search process using the GPD
12/31/2019	Approximate date of submission of the systematic review

## PLANS FOR UPDATING THE REVIEW

This review will be updated every 4 years, under the primary responsibility of Cynthia Lum, unless all authors agree that another author takes primary responsibility. The updated review is contingent on availability and funding.

## AUTHOR DECLARATION

### Authors’ responsibilities

By completing this form, you accept responsibility for preparing, maintaining, and updating the review in accordance with the Campbell Collaboration policy. Campbell will provide as much support as possible to assist with the preparation of the review.

A draft review must be submitted to the relevant Coordinating Group within 2 years of protocol publication. If drafts are not submitted before the agreed deadlines, or if we are unable to contact you for an extended period, the relevant Coordinating Group has the right to deregister the title or transfer the title to alternative authors. The Coordinating Group also has the right to deregister or transfer the title if it does not meet the standards of the Coordinating Group and/or Campbell.

You accept responsibility for maintaining the review in light of new evidence, comments and criticisms, and other developments, and updating the review at least once every 5 years, or, if requested, transferring responsibility for maintaining the review to others as agreed with the Coordinating Group.

### Publication in the Campbell Library

The support of the Coordinating Group in preparing your review is conditional upon your agreement to publish the protocol, finished review, and subsequent updates in the Campbell Library. Campbell places no restrictions on publication of the findings of a Campbell systematic review in a more abbreviated form as a journal article either before or after the publication of the monograph version in Campbell Systematic Reviews. Some journals, however, have restrictions that preclude publication of findings that have been, or will be, reported elsewhere and authors considering publication in such a journal should be aware of possible conflict with the publication of the monograph version in Campbell Systematic Reviews. Publication in a journal after publication or in press status in Campbell Systematic Reviews should acknowledge the Campbell version and include a citation to it. Note that systematic reviews published in Campbell Systematic Reviews and coregistered with Cochrane may have additional requirements or restrictions for copublication. Review authors accept responsibility for meeting any co‐publication requirements.

## Figures and Tables

**Figure A1:**
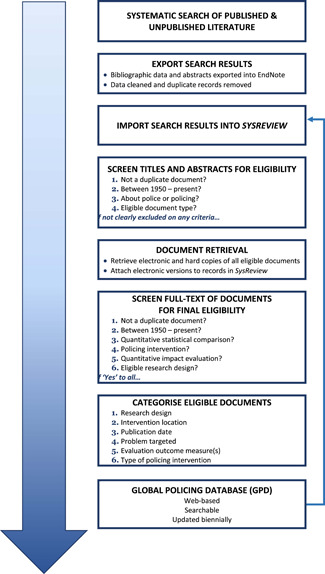
The GPD Flowchart [Color figure can be viewed at wileyonlinelibrary.com]

## Footnotes

1 While the GPD data extends back to 1950, to date full‐text documents have only been screened back to 2003. For this systematic review, the authors believe the use of the GPD is justified, as the earliest recorded evaluation for BWCs according to Lum et al. was Goodall (2007). Additionally, per the GPD search protocol, grey literature will also be searched (see Appendix B).

2 See https://bwctta.com/resources/bwc‐resources/impacts‐bwcs‐use‐force‐directory‐outcomes and https://bwctta.com/resources/bwc‐resources/impact‐bwcs‐citizen‐complaints‐directory‐outcomes.

## Appendix A GPD systematic search strategy

### Search terms

To ensure optimum sensitivity and specificity, the GPD search strategy utilizes a combination of free‐text and controlled vocabulary search terms. Because controlled vocabularies and search capabilities vary across databases, the exact combination of search terms and field codes are adapted to each database. Final search syntax for each location will be reported in the final review.

The free‐text search terms for the GPD are provided in Table A1 and are grouped by substantive (i.e., some form of policing) and evaluation terminology. Although the search strategy across search locations may vary slightly, the search follows a number of general rules:

- Search terms will be combined into search strings using Boolean operators “AND” and “OR”. Specifically, terms within each category will be combined with “OR” and categories will be combined with “AND”. For example: (police OR policing OR “law#enforcement”) AND (analy* OR ANCOVA OR ANOVA OR …).
- Compound terms (e.g., law enforcement) will be considered single terms in search strings by using quotation marks (i.e., “law*enforcement”) to ensure that the database searches for the entire term rather than separate words.
- Wild cards and truncation codes will be used for search terms with multiple iterations from a stem word (e.g., evaluation, evaluate) or spelling variations (e.g., evaluat* or randomi#e).
- If a database has a controlled vocabulary term that is equivalent to “POLICE”, we will combine the term in a search string that includes both the policing and evaluation free‐text search terms. This approach will ensure that we retrieve documents that do not use policing terms in the title/abstract but have been indexed as being related to policing in the database. An example of this approach is the following search string: (((SU: “POLICE”) OR (TI,AB,KW: police OR policing OR “law*enforcement”)) AND (TI,AB,KW: intervention* OR evaluat* OR compar* OR …)).
- For search locations with limited search functionality, we will implement a broad search that uses only the policing free‐text terms.
- Multidisciplinary database searches will be limited to relevant disciplines (e.g., include social sciences but exclude physical sciences).
- Search results will be refined to exclude specific types of documents that are not suitable for systematic reviews (e.g., newspapers, front/back matter, book reviews).

**Table A1:** Free‐text search terms for the GPD systematic search

Policing search terms	Evaluation search terms			
police policing “law*enforcement” constab* detective* sheriff*	analy* ANCOVA ANOVA “ABAB design” “AB design” baseline causa* “chi#square” coefficient* “comparison condition*” “comparison group*” “control condition*” “control group*” correlat* covariat* “cross#section*”	data effect* efficacy eval* experiment* hypothes* impact* intervent* interview* longitudinal MANCOVA MANOVA “matched group” measure* “meta‐analy*” “odds#ratio*	outcome* paramet* “post‐test” posttest “post test” predict* “pre‐test” pretest program* “propensity score*” quantitative “quasi#experiment*” questionnaire* random* RCT regress*	result* “risk#ratio*” sampl* “standard deviation*” statistic* studies study survey* “systematic review*” “t#test*” “time#series” treatment* variable* variance

### Search locations

To reduce publication and discipline bias, the GPD search strategy adopts an international scope and involves searching for literature across a number of disciplines (e.g., criminology, law, political science, public health, sociology, social science, and social work). The search captures a comprehensive range of published (i.e., journal articles, book chapters, books) and unpublished literature (e.g., working papers, governmental reports, technical reports, conference proceedings, dissertations) by implementing a search strategy across bibliographic/academic, grey literature, and dissertation databases or repositories.

We note that there is a substantial overlap of the content coverage between many of the databases. Therefore, we have used the *Optimal Searching of Indexing Databases* (OSID) computer program (Neville & Higginson, 2014) to analyze the content crossover for all databases that have accessible content coverage lists. OSID analyses the content coverage and creates a search location solution that provides the most comprehensive coverage via the least number of databases. For example, if the content for the set of databases seen in Figure A1 were imported, OSID would provide a solution that entails searching only databases 3 and 4 because the content covered by databases 1 and 2 is covered by database 4. Another advantage of using OSID when designing a search strategy is the reduction in the number of duplicates that would need to be removed prior to the screening phase. Databases with >10 unique titles were being searched in full, whereas for databases with ≤10 unique titles, we searched only the unique titles and any nonserial content (e.g., reports and conference proceedings). Where a modified search of a database would be more labor intensive than a full search and export results, conducted a full search of the database. The final search locations and solution are reported in Table A2.

**Table A2:** GPD search locations and protocol (January 1st 1950 to December 2018)

Indexed and academic databases		Content coverage fed into osid?	Full or modified search?	Search modifications
ProQuest	Criminal Justice	Yes	Full	None.
	Dissertation and Theses Database Global	Not Available	Modified	Social Sciences subset.
	Political Science	Yes	Full	None.
	Periodical Archive Online	Yes	Full	None.
	Research Library	Yes	Modified	Social Sciences subset.
	Social Science Journals	Yes	Full	None.
	Sociology	Yes	Modified	Search 2 unique journal titles and nonserial content only.
	Applied Social Sciences Index and Abstracts	Yes	Full	None.
	International Bibliography of the Social Sciences	Yes	Full	None.
	Public Affairs Information Service	Yes	Full	None.
	Social Services Abstracts	Yes	Modified	Search 5 unique journal titles and nonserial content only.
	Sociological Abstracts	Yes	Full	None.
	Worldwide Political Sciences Abstracts	Yes	Modified	Search 9 unique journal titles and nonserial content only.
EBSCO	Academic Search Premier	Yes	Full	None.
	Criminal Justice Abstracts	Yes	Full	None.
	EconLit	Yes	Full	None.
	MEDLINE with Full‐Text	Yes	Full	None.
	Social Sciences Full‐Text	Yes	Full	None.
OVID	International Political Science Abstracts	Not Available	Full	None.
	PsycARTICLES	Yes	Modified	Search 4 unique journal titles only.
	PsycEXTRA	Not Available	Full	None.
	PsycINFO	Yes	Full	None.
	Social Work Abstracts	Not Available	Full	None.
Web of Science	Current Contents Connect – Social and Behavioural Sciences Edition	Yes	Modified	Search 1 unique journal title and nonserial content only.
	Book Citation Index (Social Sciences and Humanities)	Not Available	Full	None.
	Conference Proceedings Citation Index (Social Sciences and Humanities)	Not Available	Full	None.
	Social Science Citation Index	Yes	Full	None.
Informit	Australian Attorney‐General Information Service	Yes	Full	None.
	Australian Criminology Database (CINCH)	Yes	Full	None.
	Australian Federal Police Database	Yes	Full	None.
	Australian Public Affairs Full‐Text	Yes	Full	None.
	DRUG	Yes	Full	None.
	Health & Society Database	Yes	Modified	Search unique journal titles and nonserial content only.
	Humanities and Social Sciences Collection	Yes	Full	None.
Gale‐Cengage	Expanded Academic ASAP	Yes	Full	None.
Standalone and open access databases	Cambridge Journals Online	Yes	Modified	Search 4 unique journal titles in Law and Political Science collections and a full search of Social Studies collection.
	Directory of Open Access Journals	Yes	Full	None.
	HeinOnline	Yes	Modified	Law Journals Online collection only.
	JSTOR	Yes	Modified	Search unique titles across the Law, Political Science, Public Health, Public Policy, Social Work and Sociology collections only. The Criminal Justice collection had no unique content and so will be excluded from the search. Only 10% of the content in this database have abstracts and a full‐text search returns > 250,000 results because of inability to construct complex search strings. Therefore, a modified search of the unique titles across these collections will be more pragmatic than a full search of the database.
	Oxford Scholarship Online	Yes	Full	None.
	Sage Journals Online and Archive (Sage Premier)	Yes	Modified	Search 5 unique journal titles and nonserial content only.
	ScienceDirect	Yes	Full	None.
	SCOPUS	Yes	Full	None.
	SpringerLink	Yes	Full	Although this database has low uniqueness when combined with the full set of databases, a full search using only the policing search terms will be more pragmatic than a modified search on unique titles because of the restricted search functionality of this database.
	Taylor & Francis Online	Yes	Modified	Although this database has low uniqueness when combined with the full set of databases, a full search using only the policing search terms will be more pragmatic than a modified search on unique titles because of the restricted search functionality of this database.
	Wiley Online Library	Yes	Full	None.
	California Commission on Peace Officer Standards & Training Library	No	Full	None.
	Cochrane Library	No	Full	None.
	CrimeSolutions.gov	No	Full	None.
	Database of Abstracts of Reviews of Effectiveness (DARE)	No	Full	None.
	FBI – The Fault (Reports and Publications)	No	Full	None.
	Evidence‐Based Policing Matrix	No	Full	None.
	International Initiative for Impact Evaluation Database (3ie)	No	Full	None.
	National Criminal Justice Reference Service	No	Full	None.
	Safety Lit Database	No	Full	None.
	Australian Institute of Criminology	No	Full	None.
	Bureau of Police Research and Development (India)	No	Full	None.
	Canadian Police Research Catalogue	No	Full	None.
	Centre for Problem‐Oriented Policing	No	Full	None.
	College of Policing (including POLKA and Crime Reduction Toolkit)	No	Full	None.
	European Police College (CEPOL)	No	Full	None.
	Evidence for Policy and Practice Information and Coordinating Centre	No	Full	None.
	National Research Institute of Police Science (Japanese)	No	Full	None.
	Office of Community Oriented Policing Services	No	Full	None.
	Police Executive Research Forum (US)	No	Full	None.
	Police Foundation (US)	No	Full	None.
	Tasmania Institute of Law Enforcement Studies (Australia)	No	Full	None.
	Policing Online Information System (POLIS, Europe)	No	Full	None.
	Scottish Institute for Policing Research	No	Full	None.
	Centre of Excellence in Policing and Security (Australian, now archived)	No	Full	None.

## Appendix B GPD systematic compilation strategy

### Inclusion criteria

Each record captured by the GPD systematic search must satisfy all inclusion criteria to be included in the GPD: timeframe, intervention, and research design. There are no restrictions applied to the types of outcomes, participants, settings or languages considered eligible for inclusion in the GPD.

#### Types of interventions

Each document must contain an impact evaluation of a policing intervention. We define a policing intervention is some kind of a strategy, program, technique, approach, activity, campaign, training, directive, or funding /organizational change that involves the police in some way (other agencies or organizations can be involved). Police involvement is broadly defined as

- Police initiation, development or leadership
- Police are recipients of the intervention or the intervention is related, focused or targeted to police practices
- Delivery or implementation of the intervention by police

#### Types of study designs

The GPD includes quantitative impact evaluations of policing interventions that utilize randomized experimental (e.g., RCTs) or quasi‐experimental evaluation designs with a valid comparison group that does not receive the intervention. The GPD includes designs where the comparison group receives “business‐as‐usual” policing, no intervention or an alternative intervention (treatment‐treatment designs).

The specific list of research designs included in the GPD are as follows:

- Systematic reviews with or without meta‐analyses
- Crossover designs
- Cost‐benefit analyses
- Regression discontinuity designs
- Designs using multivariate controls (e.g., multiple regression)
- Matched control group designs with or without pre‐intervention baseline measures (propensity or statistically matched)
- Unmatched control group designs with pre‐post intervention measures which allow for difference‐in‐difference analysis
- Unmatched control group designs without pre‐intervention measures where the control group has face validity
- Short interrupted time‐series designs with a control group (less than 25 preintervention and 25 postintervention observations (Glass, 1997))
- Long interrupted time‐series designs with or without a control group (≥25 pre‐ and post‐intervention observations (Glass, 1997))
- Raw unadjusted correlational designs where the variation in the level of the intervention is compared to the variation in the level of the outcome

The GPD excludes single group designs with pre‐ and post‐intervention measures as these designs are highly subject to bias and threats to internal validity.

### Systematic screening

To establish eligibility, records captured by the GPD search progress through a series of systematic stages which are summarised in Figure A1, with additional detail provided in the following subsections.

All research staff working on the GPD undergo standardized training before beginning work within any of the stages detailed below. Staff then complete short training simulations to enable an assessment of their understanding of the GPD protocols and highlight any areas for additional training. In addition, random samples of each staff's work are regularly cross‐checked to ensure adherence to protocols. Disagreements between staff are mediated by either the project manager or GPD chief investigators.

#### Title and abstract screening

After removing duplicates, the title and abstract of record captured by the GPD systematic search is screened by trained research staff to identify potentially eligible research that satisfy the following criteria:

- Document is dated between 1950 to present
- Document is unique (i.e., not a duplicate)
- Document is about police or policing
- Document is an eligible document type (e.g., not a book review)

Records are excluded if the answer to any one of the criteria is unambiguously “No,” and will be classified as potentially eligible otherwise. Records classified as potentially eligible progress to full‐text document retrieval and screening stages.

#### Full‐text eligibility screening

Wherever possible, a full‐text electronic version of eligible records will be imported into SysReview. For records without an electronic version, a hardcopy of the record will be located to enable full‐text eligibility screening. The full text of each document will be screened to identify studies that satisfy the following criteria:

- Document is dated between 1950 to present;
- Document is unique;
- Document reports a quantitative statistical comparison;
- Document reports on policing evaluation;
- Document reports in quantitative impact evaluation of a policing intervention; and
- Evaluation uses an eligible research design.

## Appendix C Coding forms and instructions

Version: July 8, 2019

Note: This is a living document that will be updated and modified during the coding process as decisions are made regarding how to handle edge cases or other refinements that are made to the coding protocol.

Coding will be done directly into a MySQL relational database using Libreoffice Base as the front‐end with detailed coding forms that reflect the coding protocol.

### Initial eligibility screening

Initial eligibility screening will be performed on all titles and abstracts identified by the bibliographic search. Two coders will independently assess whether the title and abstract suggest that the study may meet the full eligibility criteria (see protocol). Each coder will determine if the reference is “potentially eligible”, “not eligible”, a “relevant review”, or that it is “unclear”. A reference marked as unclear will be assessed by another coder. Any reference marked as “potentially eligible” by either coder will move forward to a full‐text eligibility assessment.

### Final (full‐text) eligibility screening

For full‐text screening, each document (reference) will be assessed against the four criteria of the eligibility criteria (see protocol or database coding form for Final Screening). For each criterion, answer “yes”, “no” or “uncertain”. If the coder answers “yes” to all four criteria, code the reference as “Eligible”. If the coder answers “no” to any item, code the reference as “Not Eligible”. If there are mix of “yes” and “uncertain” then another coder must make the final assessment.

Two coders will assess each reference for eligibility. Any discrepancies will be resolved through a consensus process.

### Study level coding

1. Study ID & Substudy ID: These two field uniquely identify the study and sub‐study and should correspond to a record coded at the study level. Label substudies sequentially by letter (i.e., A, B, C, …). This field cannot be left blank. Thus, if there is only one study, the substudy is “A”.
2. Author/year label. Create a unique author label for each study/substudy combination that suitable for use on a forest plot (e.g., “Ariel 2017 Denver” and “Braga et al 2018 Las Vegas”).
3. Coder's Initials: Insert your initials. This is case‐insensitive.
4. Date Modified: This is auto‐generated and is a timestamp for the last time any changes were made to this record.
5. Publication Types (check all that apply [multiple documents may be coded as part of one study]):
  1. Journal
  2. Book
  3. Book Chapter
  4. Technical report (government agency)
  5. Technical report (university/research institute)
  6. Dissertation/thesis
  7. Presentation
  8. Other

### Context of the study

6. Specific location or jurisdiction in which the study was conducted [text box, this could be a city, county, state, country such as “Tucson” or “Colombia” or “Maryland” (if conducted across the entire state)]
7. State [text box, if in the U.S., use full state name, including “District of Columbia”]
8. Country [text box, use full name of the country – “United States”, not “U.S.”]
9. Population density (population per square mile from the 2010 U.S. Census of the specific geographic area that study location is a part of)
10. Diversity Index (Calculated and added from 2010 Census information based on established diversity index formula)
11. Law enforcement agency name if known [text box].
12. Number of full‐time sworn officers in the agency in the year in which the intervention was initiated [number box, found from annual UCR LEOKA files]
13. Agency type
  1. law enforcement only
  2. law enforcement agency with correctional duties [i.e., Sheriff's agencies; requirement of the review is that officers with BWCs have law enforcement duties]
  3. regional agency that oversees multiple jurisdictions or a national agency
14. Primary university or research organization in which the study was conducted [text box, full name of university (“Florida State University”) or the research organization (“Police Executive Research Forum”)].
15. Were BWCs used in the agency prior to the study?
  1. BWCs were already in use by agency before the study began across the agency (not selective use)
  2. BWCs were already in use by agency before the study began but only by specific and very limited units/people or in a pilot testing phase
  3. Use of BWCs began very close to the time of the study or for the purposes of the study
16. If known, the year of BWC adoption by the agency [text box year]
17. In the 2 years prior to camera adoption, had this agency or jurisdiction undergone a collaborative reform, consent decree, or USDOJ review? YES/NO

### Intervention information

18. Nature of BWC use during the intervention
  1. Uniformed patrol only
  2. Specialized units only (traffic, investigative, community‐oriented, etc.)
  3. Combination uniformed patrol and specialized units
  4. Training environments (academy, in‐service)
  5. Other [text box]
19. Activation requirements for BWC use specific to the intervention condition (agency may have had different activation policies prior to the intervention, but this is specific to the intervention and study)
  1. Activated at all times with exceptions as described in official policy (bathroom breaks, hospitals, child victims)
  2. Activation policies are specified for categories of incidents and activities as described in policy (turned off at all other times)
  3. Discretionary activation (officers can choose whether to turn on/off)
  4. No activation policy
  5. Unknown/unclear activation policy
20. Month/year study intervention started
21. Month/year study intervention finished
22. Data collection start date (month/year) (this includes the date when baseline data/measures started to be collected)
23. Data collection end date (month/year)
24. Challenges to implementation (fidelity issues) [text box]

### Research methods and design

25. Unit of Analysis
  1. Officer
  2. Shift
  3. Officer‐shift combinations
  4. Enforcement groups (squads, specialized units)
  5. Police‐defined geographic areas (beats, districts, sectors)
  6. Other geographic areas (hot spots, census blocks, etc.)
  7. Other [text box]
26. Type of experimental or quasi‐experimental design
  1. RCT (randomized experiment)
  2. Cluster RCT (higher‐order units randomized)
  3. Quasi‐experiment: simple matching
  4. Quasi‐experiment: propensity score matching
  5. Quasi‐experiment: statistical adjustments for baseline features (including time trend)
  6. Quasi‐experiment: historical/cohort design
  7. Quasi‐experiment: other
  8. Eligible time series
27. How the sample was selected?
  1. All available units of analysis were used
  2. Convenience sampling: only those who volunteered were used
  3. Specific sample: Agency only wanted specific units to use BWCs and/or be involved in the study
  4. Other [text box]
28. Description of sample selection bias [text box]
29. Treatment description [text box]
30. Treatment group sample size at the start of the study [number box]
31. Control group description [text box]
32. Control group sample size at the start of the study [number box]
33. Attrition concerns described [text box]
34. A priori power calculations?
  1. Yes (explicitly stated)
  2. No (not explicitly stated; no information)

### Cochrane risk‐of‐bias

(Y = Yes; PY = Probably Yes; PN = Probably No; N = No; NI = No information)

Domain 1: Risk of bias arising from the randomization process

1.1Was the allocation sequence random?
  Y/PY/PN/N/NI
1.2Was the allocation sequence concealed until participants were enrolled and assigned to interventions?
  Y/PY/PN/N/NI
1.3Did baseline differences between intervention groups suggest a problem with the randomization process? [For quasi‐experiments, were there meaningful differences on baseline variables?]
  Y/PY/PN/N/NI
  Optional: Were there violations to the randomization? That is, were members of the control group exposed to the treatment and/or members of the treatment group who were not exposed to the treatment?
  Y/PY/PN/N/NI
Optional: What is the predicted direction of bias arising from the randomization process? [For quasi‐experimental designs, what is the predicted direction of bias of the selection process?]

Favors Experimental/Favors Comparator/

Towards Null/Away From Null/Unpredictable

Domain 2: Risk of bias due to deviations from the intended interventions (effect of assignment to intervention)

2.6Was an appropriate analysis used to estimate the effect of assignment to intervention?

Y/PY/PN/N/NI

2.7Was there potential for a substantial impact (on the result) of the failure to analyze participants in the group to which they were randomized?

Y/PY/PN/N/NI

Domain 5: Risk of bias in selection of the reported result

Is [Are] the numerical result [results] being assessed likely to have been selected, on the basis of the results, from…

5.2… multiple outcome measurements (e.g. scales, definitions, time points) within the outcome domain?

Y/PY/PN/N/NI

5.3… multiple analyses of the data?

Y/PY/PN/N/NI

Optional: What is the predicted direction of bias due to selection of the reported result?

Favors Experimental/Favors Comparator/

Towards Null/Away From Null/Unpredictable

### Outcome level coding

Each eligible dependent variable or measure will be coded as a separate record. The Primary Key that is the unique identifier for each record in the outcome table (in the database, this table is named "3_Outcome") and is the combination of the following fields: StudyID, SubStudyID, OutcomeID, and CoderID. The StudyID and SubStudyID.

1. Study ID & Substudy ID: These two fields uniquely identify the study and sub‐study and should correspond to a record coded at the study level (see study level coding instructions).
2. Outcome ID: This field uniquely identifies the outcome associated with this effect size. This should correspond to a record coded at the outcome level (see outcome level coding instructions).
3. Outcome label. Type a label that describes this outcome. Ideally, this should match what is used in the text unless additional clarification is needed.
4. Coder's Initials.
5. Date Modified: This is auto‐generated and is a timestamp for the last time any changes were made to this record.
6. Construct measured by this outcome
  1. Officer behavior
    - officer use of force
    - complaints against officers
    - arrest and citation behavior
    - proactive activities
  2. Civilian behavior
    - compliance with police commands as measured by resisting arrest
    - assaults against officers (which may overlap with 2a, above)
    - criminal or disorderly conduct
    - willingness to call the police or cooperate in an investigation
7. Nature of outcome data
  1. Dichotomous
  2. Discrete ordinal scaled measure (<10 categories)
  3. Discrete ordinal scaled measure (10+ categories)
  4. Count
  5. Ratios
  6. Continuous measures
  7. Unclear
8. Unit‐of‐measurement
  1. Officer
  2. Citizen
  3. Incident
  4. Shift
  5. Police unit
  6. Time period
  7. Geographic area
  8. Unclear

Cochrane Risk‐of‐Bias Domain 3: Missing outcome database

3.1Were data for this outcome available for all, or nearly all, participants randomized?

Y/PY/PN/N/NI

3.2If N/PN/NI to 3.1: Is there evidence that the result was not biased by missing outcome data?

Y/PY/PN/N/NI

3.3If N/PN to 3.2: Could missingness in the outcome depend on its true value?

Y/PY/PN/N/NI

3.4If N/PN to 3.2: Could missingness in the outcome depend on its true value?
  Y/PY/PN/N/NI
  Optional: What is the predicted direction of bias due to missing outcome data?
    NA

Favors experimental
Favors comparator
Towards the null
Away from the null
Unpredictable

Cochrance Risk‐of‐Bias Domain 4: Risk of bias in measurement of the outcome

4.2Could measurement or ascertainment of the outcome have differed between intervention groups?

Y/PY/PN/N/NI

4.3If N/PN/NI 4.2: Were outcome assessors aware of the intervention received by study participants?

Y/PY/PN/N/NI

4.4If Y/PY/NI to 4.3: Could assessment of the outcome have been influenced by knowledge of intervention received?

Y/PY/PN/N/NI

4.5If Y/PY/NI to 4.4: Is it likely that assessment of the outcome was influenced by knowledge of intervention received?

Y/PY/PN/N/NI

Optional: What is the predicted direction of bias in the measurement of the outcome?

Favors Experimental/Favors Comparator/

Towards Null/Away From Null/Unpredictable

### Effect size level coding

Each unique codeable effect for an eligible outcome should be coded as a separate record. The Primary Key that is the unique identifier for each record in the effect size table (in the database, this table is named “4_EffectSize”) is the combination of the following fields: StudyID, SubStudyID, OutcomeID, ESID, and Coder. The StudyID and SubStudyID should uniquely identify which study/substudy is being coded. The OutcomeID indicates which outcome coded at the outcome level is associated with this effect.

The coding form has input fields for nine different ways to compute an effect size. There are times when a unique effect can be computed in more than one way. Select the method that is most accurate. For example, a study may report both means, standard deviations, and sample size information along with an independent t‐test associated with these means. Both the former and latter can be used to compute the effect size and should produce the same value. However, the t‐test, unless reported to 3 or more digits, is likely to be less precise due to rounding error than the raw means and standard deviations.

In contrast to the above, the same outcome may be analyzed in different ways that would produce different effect sizes. For example, a study may report the raw means and *t*‐test but also report a regression model with a treatment dummy code that adjusts for baseline covariates. In such a situation, code two effect sizes, one based on the means and one on the regression model (i.e., method 7 on the coding form). These should be coded as separate records in the database.

1. Study ID and substudy ID: These two fields uniquely identify the study and sub‐study and should correspond to a record coded at the study level (see study level coding instructions).
2. Outcome ID: This field uniquely identifies the outcome associated with this effect size. This should correspond to a record coded at the outcome level (see outcome level coding instructions).
3. Effect Size ID: Assign each effect size for a given StudyID+SubStudyID combination a unique number, starting at 1. For example, if a Study/SubStudy has four effect sizes, these should be numbered 1, 2, 3, 4 in this field.
4. Coder's Initials: Insert your initials. This is case‐insensitive.
5. Date Modified: This is auto‐generated and is a timestamp for the last time any changes were made to this record.
6. Page number. Indicate the page number where the effect size data can be found.
7. Notes: Use this text box to indicate any notes you have recording this effect size. Use liberally. If you are uncertain about how anything is coded, please specify this in the note. Also, if you computed the effect size by hand, include relevant information so that another coder could replicate your computations in this field (e.g., insert R code if applicable).
8. Description of the timing for the effect size. Use this text box to describe the timing for the effect size. For example, the data for the effect size may reflect a 6‐month period following the start of the use of BWCs.
9. Timing for the effect size. Indicate if this effect size is measured at baseline (or pretest). For all effect sizes after the start of the use of BWC, select the post‐test 1, 2, 3, or 4 sequentially (e.g., the first post‐test is 1, next is 2, etc.).
  1. Baseline
  2. Post‐test 1
  3. Post‐test 2
  4. Post‐test 3
  5. Post‐test 4
10. Direction of effect size. Indicate if the direct for the effect favors the treatment or control. If you select “neither” then the effect size must equal 0.00. If you select “unclear” this effect size will not be used unless the direct of effect can be resolved.
  1. Treatment
  2. Control
  3. Neither
  4. Unclear
11. Effect size adjusted for baseline variables (e.g., effect size based on the regression model that includes baseline variables)
  1. Yes
  2. No
  3. Unclear
12. Is the effect reported as significant at *P* <= .05?
  1. Yes
  2. No
  3. Not tested
  4. Unclear
13. Unit‐of‐analysis for this effect (i.e., what does the sample size reflect)
  1. Officer
  2. Incident
  3. Shift
  4. Other (specify: __________)
14. Clustered or nested data for this effect?
  1. Yes
  2. No
  3. Unclear
15. If this is a clustered effect, does the study report the ICC (intra‐class correlation, the variance of the random intercept in a mixed‐effects model)?
16. Method used to compute the effect size
  1. Means and standard deviations
  2. Means and standard errors
  3. the t‐test (between two means)
  4. Frequencies (success/failure)—could be represented as a 2 by 2 (treatment/control by success/failure or some other binary outcome)
  5. Proportions (success/failure)—proportion of successes or failures in each group (could be converted to a 2 by 2 frequency table using the sample sizes)
  6. Count or rate (check with Dave Wilson before using this one)
  7. OLS/HLM regression. Results from a regression model with a continuous type‐dependent variable (OLS type regression, etc.). Treatment/control must be reflected by a dummy variable (e.g., 0/1).
  8. Logistic regression. Results from a logistic regression model. Treatment/control must be reflected by a dummy variable (e.g., 0/1).
  9. Hand calculated. This is for more complex situations where you, for example, use the online effect size calculator or computed the effect size using R.
17. Treatment group sample size (needed for all methods of computing effect sizes)
18. Control group sample size (needed for all methods of computing effect sizes)
  Method 1
19. Treatment group mean
20. Control group mean
21. Treatment group standard deviation
22. Control group standard deviation
  Method 2
23. Treatment group mean
24. Control group mean
25. Treatment group standard error
26. Control group standard error
  Method 3
27. t‐value (t‐test from an independent t comparing two means)
28. *P*‐value from above (only needed if t‐value is not available Method 4
29. Frequency of failures (or successes) in treatment group (must be a fraction of sample size)
30. Frequency of failures (or successes) in control group (must be a fraction of sample size)
  Method 5
31. Proportion of failures (or successes) in treatment group (must be a proportion of sample size)
32. Proportion of failures (or successes) in control group (must be a proportion of sample size)
  Method 6
33. Counts of events (or event rate) in treatment group
34. Counts of events (or event rate) in control group Method 7
35. Unstandardized regression coefficient for treatment dummy variable (B) (OLS or similar regression)
36. Standard error for the unstandardized regression coefficient for treatment dummy
37. Standardized regression coefficient
38. Standard deviation for the dependent variable
  Method 8
39. Unstandardized regression coefficient for treatment dummy variable (B) (logistic regression)
40. Standard error for the unstandardized regression coefficient for treatment dummy
41. Odds‐ratio
  Method 9
42. Hand calculated Cohen's d effect size
43. Hand calculated variance (standard error squared) for Cohen's d effect size
